# Identification of necroptosis-related genes in Parkinson’s disease by integrated bioinformatics analysis and experimental validation

**DOI:** 10.3389/fnins.2023.1097293

**Published:** 2023-05-22

**Authors:** Cheng Lei, Zhou Zhongyan, Shi Wenting, Zhang Jing, Qin Liyun, Hu Hongyi, Yan Juntao, Ye Qing

**Affiliations:** ^1^Department of Tuina, Longhua Hospital, Shanghai University of Traditional Chinese Medicine, Shanghai, China; ^2^Cardiovascular Research Laboratory, Longhua Hospital, Shanghai University of Traditional Chinese Medicine, Shanghai, China; ^3^Department of Neurology, Longhua Hospital, Shanghai University of Traditional Chinese Medicine, Shanghai, China; ^4^Department of Tuina, Yueyang Hospital of Integrated Traditional Chinese Medicine and Western Medicine, Shanghai University of Traditional Chinese Medicine, Shanghai, China

**Keywords:** parkinson’s disease, programmed cell, death, necroptosis, inflammation, integrated bioinformatics analysis, immune infiltration, macrophage

## Abstract

**Background:**

Parkinson’s disease (PD) is the second most common neurodegeneration disease worldwide. Necroptosis, which is a new form of programmed cell death with high relationship with inflammation, plays a vital role in the progression of PD. However, the key necroptosis related genes in PD are not fully elucidated.

**Purpose:**

Identification of key necroptosis-related genes in PD.

**Method:**

The PD associated datasets and necroptosis related genes were downloaded from the GEO Database and GeneCards platform, respectively. The DEGs associated with necroptosis in PD were obtained by gap analysis, and followed by cluster analysis, enrichment analysis and WGCNA analysis. Moreover, the key necroptosis related genes were generated by PPI network analysis and their relationship by spearman correlation analysis. Immune infiltration analysis was used for explore the immune state of PD brain accompanied with the expression levels of these genes in various types of immune cells. Finally, the gene expression levels of these key necroptosis related genes were validated by an external dataset, blood samples from PD patients and toxin-induced PD cell model using real-time PCR analysis.

**Result:**

Twelve key necroptosis-related genes including ASGR2, CCNA1, FGF10, FGF19, HJURP, NTF3, OIP5, RRM2, SLC22A1, SLC28A3, WNT1 and WNT10B were identified by integrated bioinformatics analysis of PD related dataset GSE7621. According to the correlation analysis of these genes, RRM2 and WNT1 were positively and negatively correlated with SLC22A1 respectively, while WNT10B was positively correlated with both OIF5 and FGF19. As the results from immune infiltration analysis, M2 macrophage was the highest population of immune cell in analyzed PD brain samples. Moreover, we found that 3 genes (CCNA1, OIP5 and WNT10B) and 9 genes (ASGR2, FGF10, FGF19, HJURP, NTF3, RRM2, SLC22A1, SLC28A3 and WNT1) were down- and up- regulated in an external dataset GSE20141, respectively. All the mRNA expression levels of these 12 genes were obviously upregulated in 6-OHDA-induced SH-SY5Y cell PD model while CCNA1 and OIP5 were up- and down- regulated, respectively, in peripheral blood lymphocytes of PD patients.

**Conclusion:**

Necroptosis and its associated inflammation play fundamental roles in the progression of PD and these identified 12 key genes might be served as new diagnostic markers and therapeutic targets for PD.

## 1. Introduction

Parkinson’s disease (PD) is a common degenerative disease of the central nervous system along with multisystem disorder in middle-aged and elderly people (Chong et al., 2013; Costa et al., 2023) and is the second most common neurodegenerative disease after Alzheimer’s disease (AD) in the world (Mansour et al., 2023). PD’s main clinical manifestations include static tremors, rigidity, bradykinesia and parkinsonian gait and so on (Marino et al., 2020). According to the epidemiological studies, the prevalence of PD increases with the rise of aging populations. The incidence of PD is 1.5–2% among people over 60 years old and up to 4% in people over 80 years old (Marino et al., 2020), and the global PD patients has increased from 2.5 to 6.1 million since the 1990s (Rajan and Kaas, 2022). In China, the prevalence of PD was 1.37% in people over 60 years old, and the number of people suffering from the disease has exceeded 3.62 million indicated by a community-based study (Qi et al., 2021). Recent studies have found that dyssomnia, sleep disorder, mental disorder, cognitive disorder, and somatoform autonomic dysfunction are commonly exist in PD patients and occur among different stages of motor symptoms, affecting the life quality of patients (Suzuki et al., 2022). Due to the high rate of disability and a long therapeutic procedure, PD brings heavy burden and troubles to patients and their families (Zhang et al., 2005; Kumar et al., 2010; Seol, 2010; Rajan and Kaas, 2022). Therefore, it is extremely important to study the etiology and pathogenesis of PD nowadays.

The main pathological characteristic of PD is the degeneration and necrosis of dopaminergic neurons in the substantia nigra (Mollenhauer and von Arnim, 2022), which results in the decrease of neurotransmitter dopamine (DA) synthesis as well as its amount in the synaptic cleft (Tysnes and Storstein, 2017; Marino et al., 2020). But the specific underlying mechanisms that cause the necrosis of dopaminergic neurons are not yet fully known. Necroptosis, which is a type of cellular necrosis initiated by death receptor ligands, has been discovered that might be co-related with the progression of PD (Kim et al., 2023). Moreover, necroptosis is a unique caspase-independent programed cell death with distinctive morphological features of necrosis, which is regulated by receptor interaction proteins (RIP) including RIP1 and RIP3 and their downstream signaling molecules mixed-lineage kinase domain-like protein (MLKL), and specifically blocked by the small molecule compound, necrostatin-1 (Nec-1) (Zhang and Liu, 2013; Fayaz et al., 2014; Zhang et al., 2022; Kim et al., 2023). Inflammation and immune dysregulation are fundamental pathophysiological features of PD and contribute to its progression (Tansey et al., 2022). However, the pathological role of necroptosis in the etiology of PD still needs to be clarified.

**Figure 1:**
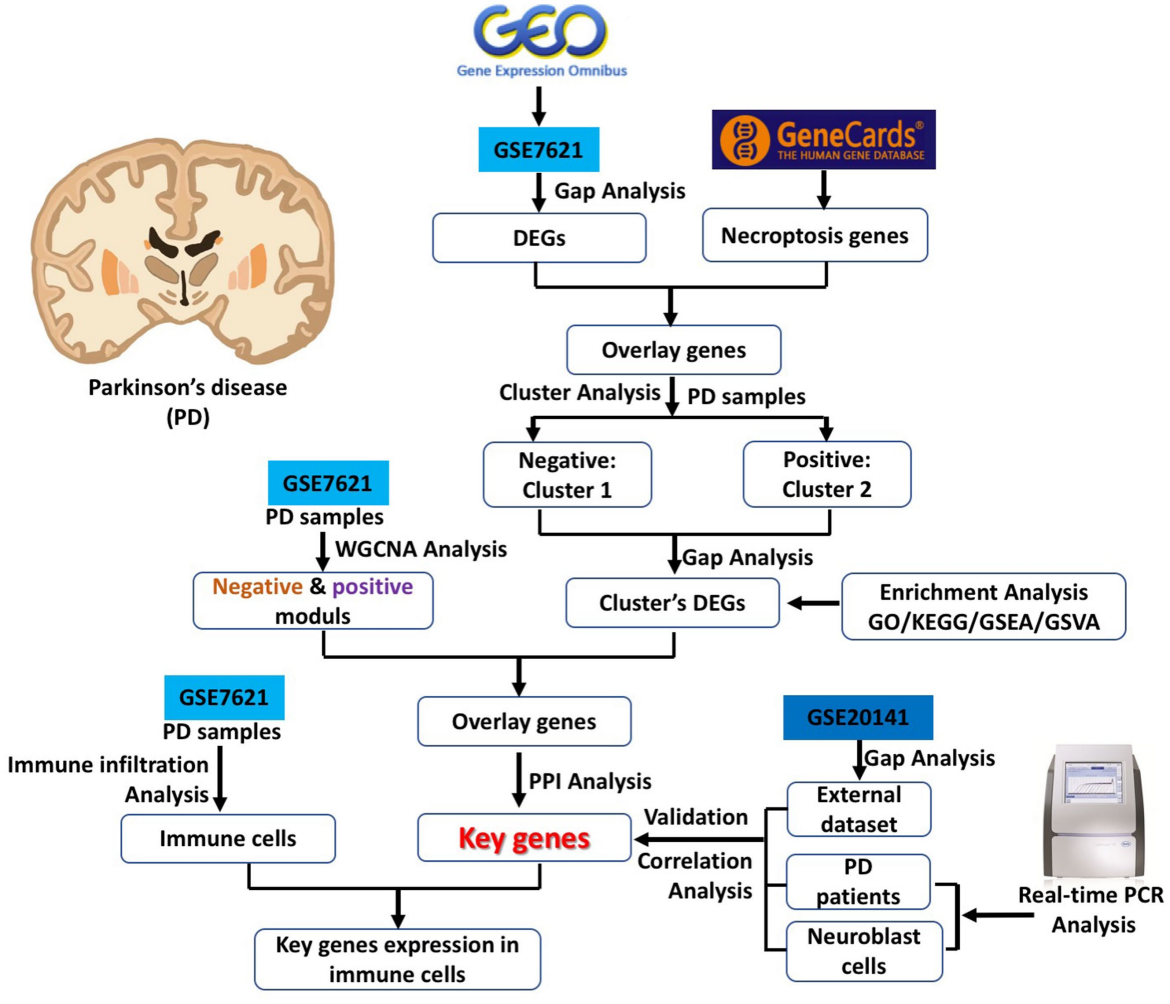
Research strategy of the current study.

In the current study, we downloaded PD related datasets from the GEO database and analyzed the associate of necroptosis-related genes between PD and control brain samples by gap analysis, cluster analysis, enrichment analysis, weighted gene co-expression network analysis (WGCNA), protein–protein interaction (PPI) networks analysis and correlation analysis according to previous studies (Zhou et al., 2020a,b; Zhao et al., 2021). Finally, several key necroptosis-related differentially expression genes (DEGs) are identified, and their interaction with immune cells was also analyzed. In addition, the mRNA expression of these key genes were verified by external dataset and real-time PCR analysis both in blood samples from control and PD patients and toxin-induced neuroblast cell PD model. The current research strategy is referred as Figure 1, and this study might provide a meaningful basis for exploring the molecular pathogenesis, diagnostic markers and drug development of PD.

## 2. Materials and methods

### 2.1. Source of data

The gene expression data were extracted from the GEO Database via the GEO query package (Davis and Meltzer, 2007). We downloaded the Parkinson’s disease-related datasets GSE7621 (Lesnick et al., 2007) and GSE20141 (Zheng et al., 2010), which are all from *Home sapiens* and generated from GPL570 data platform. GSE7621 contains 25 samples with 9 and 16 samples in the control and PD groups, respectively. GSE20141 contains 18 samples with 8 and 10 samples in the control and PD groups, respectively. All samples were employed in the current study, and we normalized and standardized the data by limma package (Ritchie et al., 2015). In addition, necroptosis-related gene sets were obtained from the GeneCards database.^1^

### 2.2. Gap analysis

Gap analysis of genes in different groups was performed using the limma R package (Ritchie et al., 2015). This research set |log FC| > 1 and *p* value <0.05 as the threshold for DEGs, where DEGs were upregulated in the disease group with logFC >0 and DEGs were downregulated in the disease group with logFC <0. The results of the gap analysis were presented by the heatmaps and volcano plots drawn by gglot2 in R package (Li et al., 2022).

### 2.3. Cluster analysis

Consensus Clustering is a method for determining the number and membership of possible clusters in a dataset (microarray gene expression). We used the “Consensus Cluster Plus” R package (Wilkerson and Hayes, 2010) to perform consensus clustering on samples from the disease group of PD in the GSE7621 dataset using differentially expressed necroptosis-related genes to facilitate better differentiation between different subtypes of PD. In this process, the number of clusters was set between 2 to 9, and 80% of the total samples were drawn in 1000 replicates, clusterAlg = “pam,” distance = “euclidean.” The *t*-Distributed Stochastic Neighbor Embedding (tSNE) technique was used for visualized and analyzed efficiently (Pezzotti et al., 2017).

### 2.4. Enrichment analysis (GO/KEGG/GSEA/GSVA)

Gene Ontology (GO) analysis is a common method for conducting large-scale functional enrichment studies, including biological process (BP), molecular function (MF) and cellular component (CC). Kyoto Encyclopedia of Genes and Genomes (KEGG) is a widely used database for storing information about genomes, biological pathways, diseases and drugs (Kanehisa and Goto, 2000). GO annotation analysis and enrichment analysis of the KEGG pathway of DEGs were performed by using the Database for Annotation, Visualization and Integrated Discovery (DAVID),^2^ with a critical value of the false discovery rate (FDR) <0.05 considered to be statistically significant (Sherman et al., 2022). The results of the enrichment analysis were visualized by using the ggplot2 in R package.

To investigate the differences in biological processes between different groups, we performed a gene set enrichment analysis (GSEA) (Subramanian et al., 2005) based on datasets of gene expression profiling from PD samples in the GSE7621 dataset. GSEA is a computational method for analyzing whether a particular gene set is statistically different between two biological states and is commonly used to estimate changes in the pathway and biological process activity in samples of expression data sets. The “c2.cp.kegg.v6.2.-symbols” gene set was downloaded from the MSigDB (Liberzon et al., 2015) for GSEA, and FDR < 0.25 was considered significantly enriched.

In addition, we use the R package to perform gene set variation analysis (GSVA) (Hänzelmann et al., 2013). The single sample gene set enrichment analysis (ssGSEA) method was used to calculate the scores of the relevant pathways based on the gene expression matrix of each sample separately, and we performed differential screening on the enrichment functions (or pathways) by using the limma package (Ritchie et al., 2015).

### 2.5. WGCNA analysis

Weighted Gene Correlation Network Analysis (WGCNA) is used for identifying correlative gene modules, exploring the relationship between gene networks and phenotypes, and studying the core genes in the network (Langfelder and Horvath, 2008). A soft threshold is calculated by the pick Soft Treshold function, with 5 being the best soft threshold, followed by the construction of a scale-free network based on the soft threshold and the construction of a topological matrix, and finally the hierarchical clustering. Eigengenes were calculated by dynamically cutting the identified gene modules with the minimum number of genes 50 in the module. Inter-module correlations were constructed based on module eigengenes, and hierarchical clustering was performed to merge modules with correlations above 0.4 (Zhao et al., 2015). The generation of modules and correlations between modules and clinical features were known through spearman correlation analysis (Wang et al., 2020).

### 2.6. PPI network construction

The STRING database^3^ is a database for searching known proteins and predicted protein–protein interactions for 2031 species, containing 9.6 million proteins and 138 million protein–protein interactions (Szklarczyk et al., 2017). It contains results obtained from experimental data, text mining of PubMed abstracts, and other database data as well as results predicted by using bioinformatics methods. We constructed a protein–protein interaction (PPI) network of differentially expressed necroptosis-related genes using the STRING database with the parameter of a factor = 0.4 by default software execution. And spearman correlation analysis was employed for the relation between identified key necroptosis related genes (Wang et al., 2020).

### 2.7. Immune infiltration analysis

CIBERSORT is based on the principle of linear support vector regression to deconvolute the transcriptome expression matrix to estimate the composition and abundance of immune cells in a mixture of cells (Chen et al., 2018). We uploaded the gene expression matrix data to CIBERSORT and combined it with the LM22 eigengene matrix to screen samples with the criteria of *p* < 0.05, and finally generated the immune cell infiltration matrix. The R programming ggplot2 package was used to plot bar graphs to show the distribution of the 22 kinds of immune cells infiltrating in each sample.

### 2.8. Quantitative real-time PCR analysis

Twelve venous blood samples from clinical PD patients (*n* = 6) and healthy adults (*n* = 6) were collected. Peripheral blood lymphocytes were separated from a lymphocyte separation medium cushion (Ficoll-Paquplus, GE). Besides, toxin-induced cell model was employed for gene expression verification. The neuroblast SH-SY5Y cells (ATCC) were treated with or without 200 μM 6-Hydroxydopamine (6-OHDA) (Sigma) for 24 h, then the cells were harvested for further analysis. Total RNA was extracted using TRIZOL (Roche). The RNA concentration was determined by a UV-spectrophotometer (M200, Tecan). Reverse transcription was performed following the reverse transcript kit (Transcriptor First Strand cDNA Synthesis Kit, Roche). Quantitative PCR was performed using SYBR Green (LightCycler 480 SYBR Green I Master, Roche) based on the LightCycle 96 platform (Roche). The PCR conditions were as follows: 95°C for 30 s, followed by 45 cycles of 95°C for 5 s, 60°C for 10 s and 72°C for 60s according to our previous study (Zhou et al., 2020a, 2023). GAPDH was used as an internal reference and gene expression changes were counted by the 2^−ΔΔCt^ method. The specific primer sequences of interest genes are shown in Table 1.

**Table 1:** Sequences of the required primers in real-time PCR analysis.

Gene	Forward primer	Reverse primer
HJURP	5’-GTCCTGGGAGCCGATTCAAA-3′	5’-CAAAGGGCTTTGAGGCACTG −3′
ASGR2	5’-TGCTCCATGGTCTGCTTCAG-3′	5’-TCACACAGATGACCACCAGC-3′
CCNA1	5’-GATAACGACGGGAAGAGCGG-3′	5’-CGGTCTCCATCCCAAGTGAC-3′
FGF10	5’-TTGTAGAAGTGGCTCGCAGG-3′	5’-GGTGGGGAATAGGGGGAGAT-3′
FGF19	5’-GAACTGACTGGAGCAGGCAT-3′	5’-GACACCGGGACAGCAAGTTA-3′
NTF3	5’-TGCCAGAGCCTGCTCTTAAC-3′	5’-GATGCCACGGAGATAAGCGA-3’
OIP5	5’-CGCCCTTCCTAGTTGGCATT-3’	5’-CGGGAATCCCACAAGAACCA-3’
RRM2	5’-GCGCGGGAGATTTAAAGGC-3’	5’-ACACGGAGGGAGAGCATAGT-3’
SLC22A1	5’-CATTTTGTTTGCGGTGTTGGG-3’	5’-TTTCTCCCAAGGTTCTCGGC-3’
SLC28A3	5’-AAACGGAGTCTCCACTGCTG-3’	5’-CAAGTGGGAGGATGGAACCC-3’
WNT1	5’-TACCTCCAGTCACACTCCCC-3’	5’-TTGAGGAGTCCCCAGGTAGG-3’
WNT10B	5’-GGGTGGCTGTAACCATGACA-3’	5’-TTGTGGATTCGCATTCGTGC-3’
GAPDH	5’-CACCATCTTCCAGGAGCGAG-3’	5’-GACTCCACGACGTACTCAGC-3’

### 2.9. Statistical analysis

All data calculations and statistical analyses were performed by using R programming^4^ (version 4.1.2). For the comparison of two groups of continuous variables, the statistical significance of normally distributed variables was estimated by independent Student *t* tests, and differences between non-normally distributed variables were analyzed by the Mann–Whitney U test (i.e., Wilcoxon rank sum test). Correlation analysis was performed on the two data sets by using Spearman’s rank correlation test. All statistical *p*-values were two-sided, with *p* < 0.05 considered statistically significance.

## 3. Results

### 3.1. Identification of DEGs and necroptosis-related genes by gap analysis

**Figure 2:**
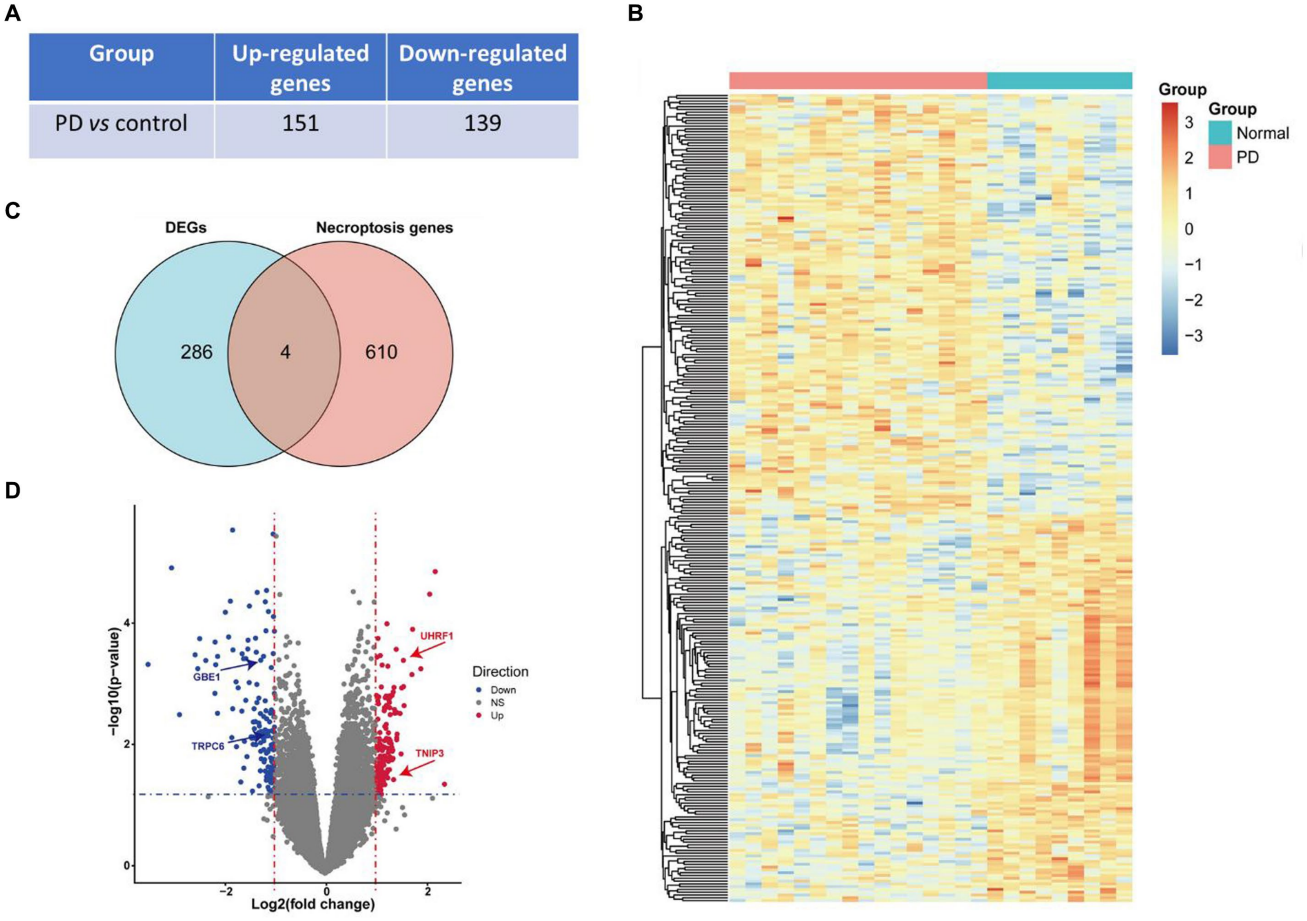
Identification of DEGs and necroptosis-related genes. **(A)** The number of up- and down- regulated genes in PD vs. control groups. **(B)** The heatmap of differentially expressed genes (DEGs) in PD vs. control groups. **(C)** Venn diagram presented 4 overlayed differentially expressed necroptosis-related genes. **(D)** Volcano plot of differentially expressed genes, in which red means up-regulated genes in the PD vs. control group while blue means down-regulated and grey means non-differentially expressed genes. The blue horizontal dotted line represents the threshold of *p* value <0.05 and the red vertical dotted line represents the threshold of |fold change| > 1. Four differentially expressed necroptosis genes, including UHRF1, GBE1, TRPC6 and TNIP3, were marked with arrows.

A total of 290 DEGs were obtained from the bioinformatic analysis of Parkinson’s disease-related dataset GSE7621 with the criterias of |logFC| > 1 and *p* value <0.05, in which 151 genes were up-regulated and 139 genes were down-regulated in PD group (Figure 2A). These up- and down- regulated genes were visualized by a heatmap (Figure 2B) and a volcano plot (Figure 2D). Moreover, 614 necroptosis-related gene sets were obtained from the GeneCards database, in which 4 genes, including ubiquitin like with PHD and ring finger domains 1 (UHRF1), 1,4-alpha-glucan branching enzyme 1 (GBE1), transient receptor potential cation channel subfamily C member 6 (TRPC6) and TNFAIP3 interacting protein 3 (TNIP3), overlayed with above DEGs (Figure 2C). And these 4 necroptosis-related genes were also marked with arrows in the volcano plot (Figure 2D).

### 3.2. Molecular classification of PD samples and DEGs based on necroptosis-related genes by cluster analysis

**Figure 3:**
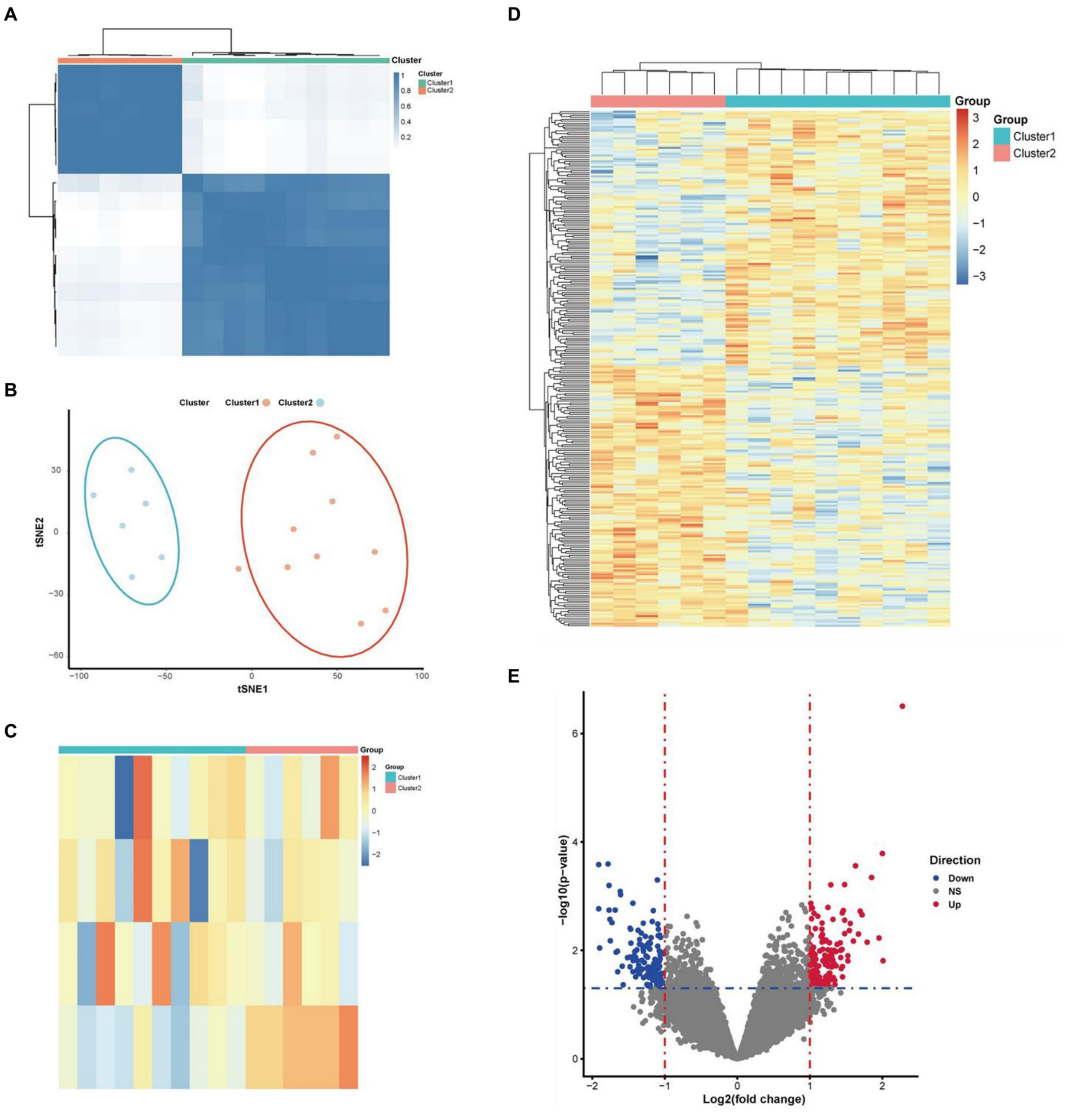
Molecular classification of PD samples and DEGs based on 4 identified differentially expressed necroptosis-related genes. **(A)** According to the guidance of four above differentially expressed necroptosis-related genes in molecular classification, Parkinson’s patients can be significantly divided into two categories. **(B)** The tSNE plot shown a clear separation of low and high necroptosis types of patients. **(C)** The heatmap shown that the patients presented low and high necroptosis phenotypes in cluster1 and cluster 2, respectively. **(E)** The volcano map shown the expression levels and connections of DEGs in these two types of Parkinson’s patients. **(D)** The heatmap shown the DEGs and their connections in these two phenotypes of Parkinson’s patients.

Concordance clustering of expressed genes in 16 PD samples was performed using above 4 differentially expressed necroptosis-related genes by the Consensus Cluster Plus package. The expressed genes were re-divided into two categories of cluster 1 and cluster 2 according to the necroptosis phenotype (Figure 3A). The tSNE plot shows that the two classes of PD samples can be significantly distinguished (Figure 3B). Visualization of differentially expressed necroptosis related genes in both patient groups revealed that patients in cluster 1 presented low necroptosis phenotype and in cluster 2 presented high necroptosis phenotype (Figure 3C). Further gap analysis was performed for these two types of PD samples with the criterias of |logFC| > 1 and *p* value <0.05. A total of 271 DEGs associated with the necroptosis phenotype were obtained and visualized using a heatmap (Figure 3D) and a volcano plot (Figure 3E).

### 3.3. Identification of both function and pathway of necroptosis-related genes in PD samples by GO, KEGG, GSEA, and GSVA enrichment analysis

**Figure 4:**
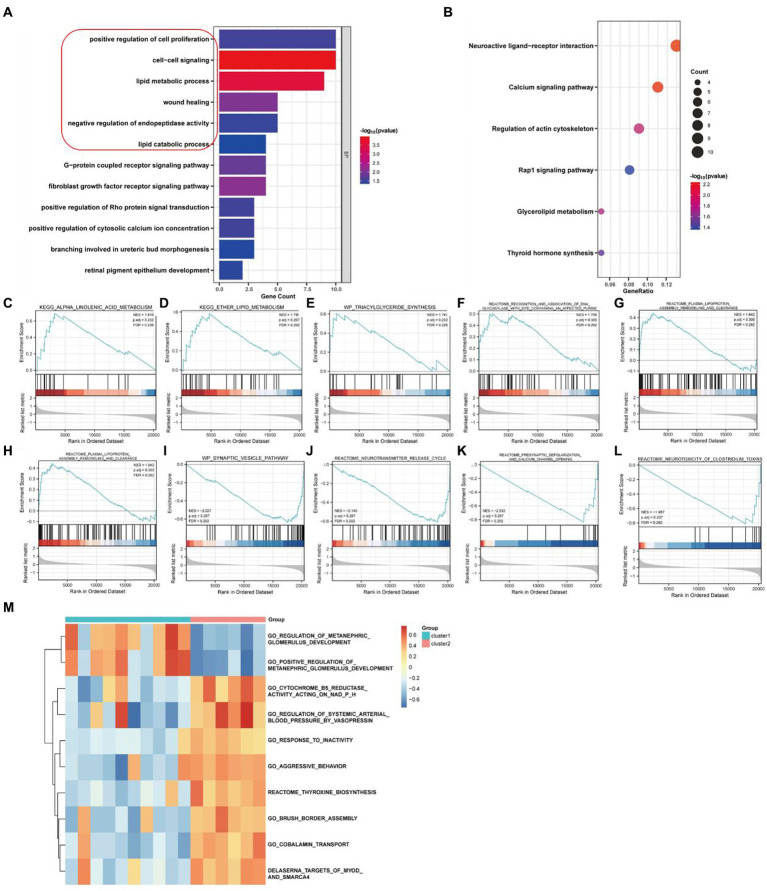
Enrichment analysis of necroptosis-related genes in PD samples. **(A)** GO enrichment analysis. **(B)** KEGG enrichment analysis. **(C–G)** Five up-regulated pathways were significantly enriched in cluster2 vs. cluster1. **(H–L)** Five down-regulated pathways were significantly enriched in cluster2 vs. cluster1 by GSEA enrichment analysis. **(M)** Pathway entries were significantly enriched in cluster1 and cluster2 by GSVA enrichment analysis.

We performed an enrichment analysis of DEGs in these two clusters of PD samples to elucidate functional differences by GO and KEGG analysis. According to GO analysis, the related biological processes are significantly enriched in entries such as positive regulation of cell proliferation, cell–cell signaling, lipid metabolic process, wound healing and negative regulation of endopeptidase activity (Figure 4A; Supplementary Table S1), while the results of KEGG analysis suggested that neuroactive ligand-receptor interaction, calcium signaling pathway, regulation of actin cytoskeleton, Rap1 signaling pathway and glycerolipid metabolism were close association (Figure 4B; Supplementary Table S2). In addition, as the result from GSEA enrichment analysis of these DEGs, five up-regulated pathways were significantly enriched in high necroptosis phenotype cluster 2 (Figures 4C–G) and five pathways were down-regulation (Figures 4H–J) respectively (Supplementary Table S3). As the results from GSVA analysis, ten selected pathways significantly enriched in both cluster 1 and cluster 2 (Figure 4M; Supplementary Table S4).

### 3.4. Generation of positive and negative modules involved in necroptosis by WGCNA analysis

**Figure 5:**
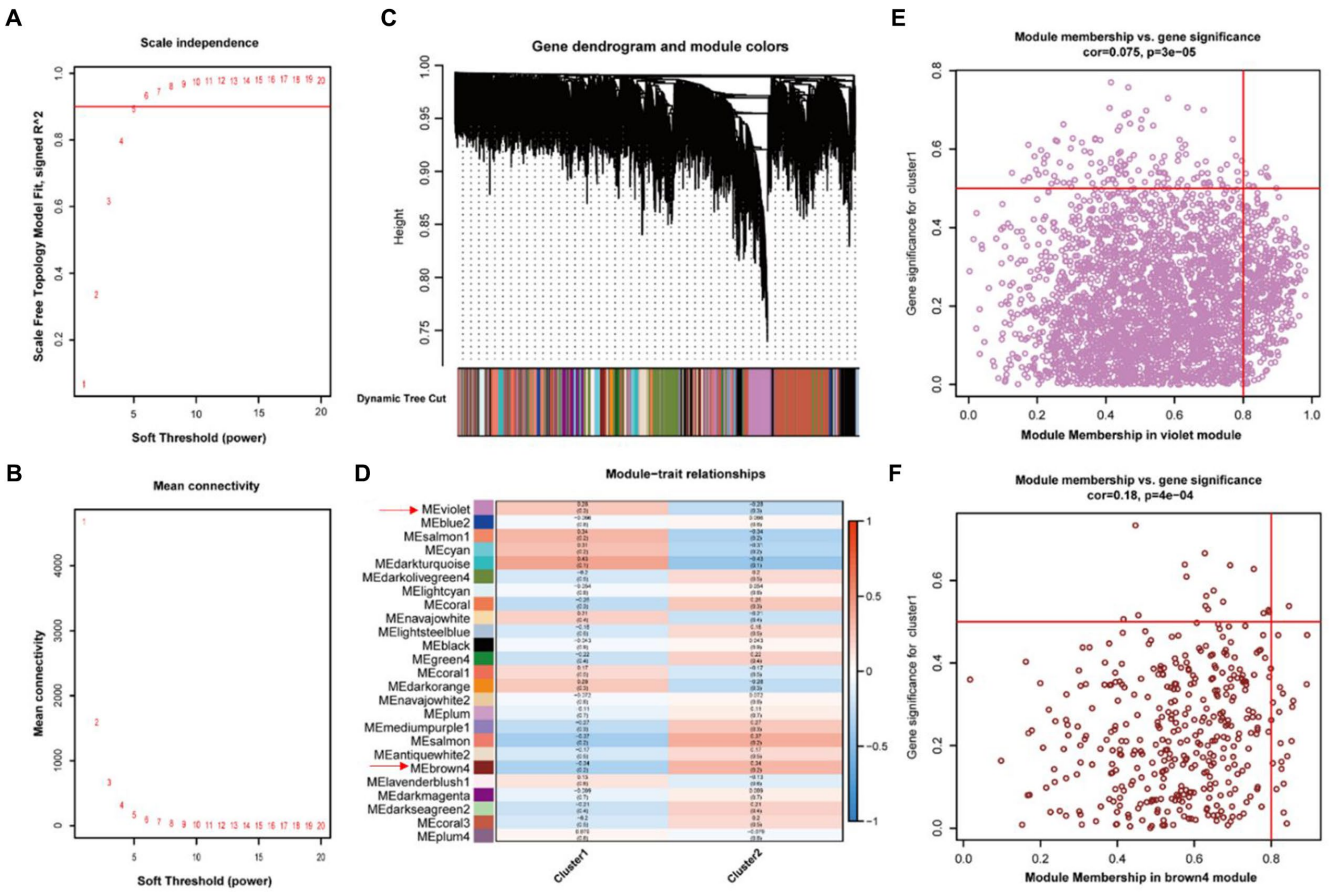
Generation of positive and negative modules involved in necroptosis by WGCNA Analysis. **(A,B)** Soft threshold screening. **(C)** Identification of gene modules by dynamic shearing tree. **(D)** Correlation analysis between modules and phenotypes. **(E)** Scatterplot of violet modules. **(F)** Scatterplot of brown4 modules.

In order to further identification of genes significantly associated with the necroptosis-related phenotype, we performed WGCNA with the criteria of 5 as the optimal soft threshold (Figures 5A,B). We performed hierarchical clustering after constructing scale-free networks and topological matrices and finally obtained 25 modules as well as correlations between modules and clinical features (Figures 5C,D). We obtained 3,091 genes in the violet module (Figure 5E), which positively regulated necroptosis, and 383 genes in the brown4 module (Figure 5F), which negatively regulated necroptosis, consequently identification of a total of 3,474 genes which were significantly associated with necroptosis.

### 3.5. Identification of 12 key necroptosis related genes in PD and their correlation analysis

**Figure 6:**
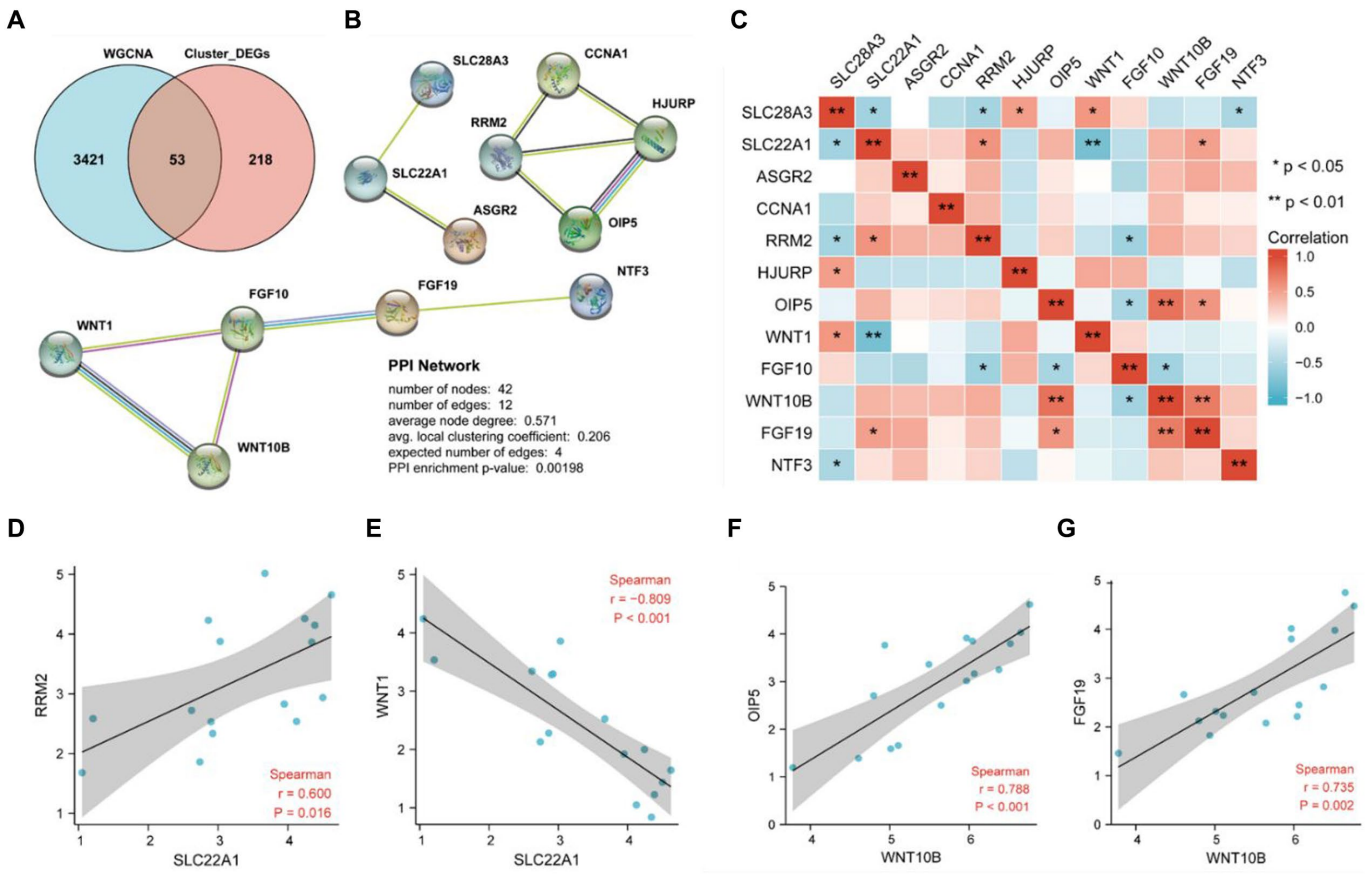
Identification of key genes by PPI network analysis and their correlation Analysis. **(A)** The 53 overlay genes between WGCNA analysis and differentially expressed necroptosis-related genes in cluster 2 were presented by Venn diagram. **(B)** Twelve key necroptosis-related genes were identified by a PPI network analysis of the 53 overlayed genes. **(C)** The heat map indicated the correlation between 12 genes by spearman correlation analysis. **(D)** Correlation analysis between SLC22A1 and RRM2. **(E)** Correlation analysis between SLC22A1 and WNT1. **(F)** Correlation analysis between WNT10B and OIP5. **(G)** Correlation analysis between WNT10B and FGF19.

The DEGs between cluster 1 and cluster 2 were intersected with the relevant module genes in WGCNA, and we obtained 53 differentially expressed necroptosis-related genes that may contribute to the different phenotypes of these two clusters of PD samples (Figure 6A). The PPI network was construct based on these 53 overlayed genes using STRING database with the criteria of coefficient = 0.4, and finally we obtained three subnetworks which contained a total of 12 key upregulated genes (Figure 6B) including asialoglycoprotein receptor 2 (ASGR2), cyclin A1 (CCNA1), fibroblast growth factor 10 (FGF10), fibroblast growth factor 19 (FGF19), holliday junction recognition protein (HJURP), neurotrophin 3 (NTF3), opa interacting protein 5 (OIP5), ribonucleotide reductase regulatory subunit M2 (RRM2), solute carrier family 22 member 1 (SLC22A1), solute carrier family 28 member 3 (SLC28A3), wnt family member 1 (WNT1) and wnt family member 10B (WNT10B). As the results from correlation analysis of these 12 key necroptosis related genes in Figures 6C–G, we found that RRM2 and WNT1 were dramatically positively and negatively correlated with SLC22A1, while WNT10B was positively correlation with both OIF5 and FGF19.

### 3.6. The M2 macrophage was the highest population among immune cells by the immuno-infiltration analysis

**Figure 7:**
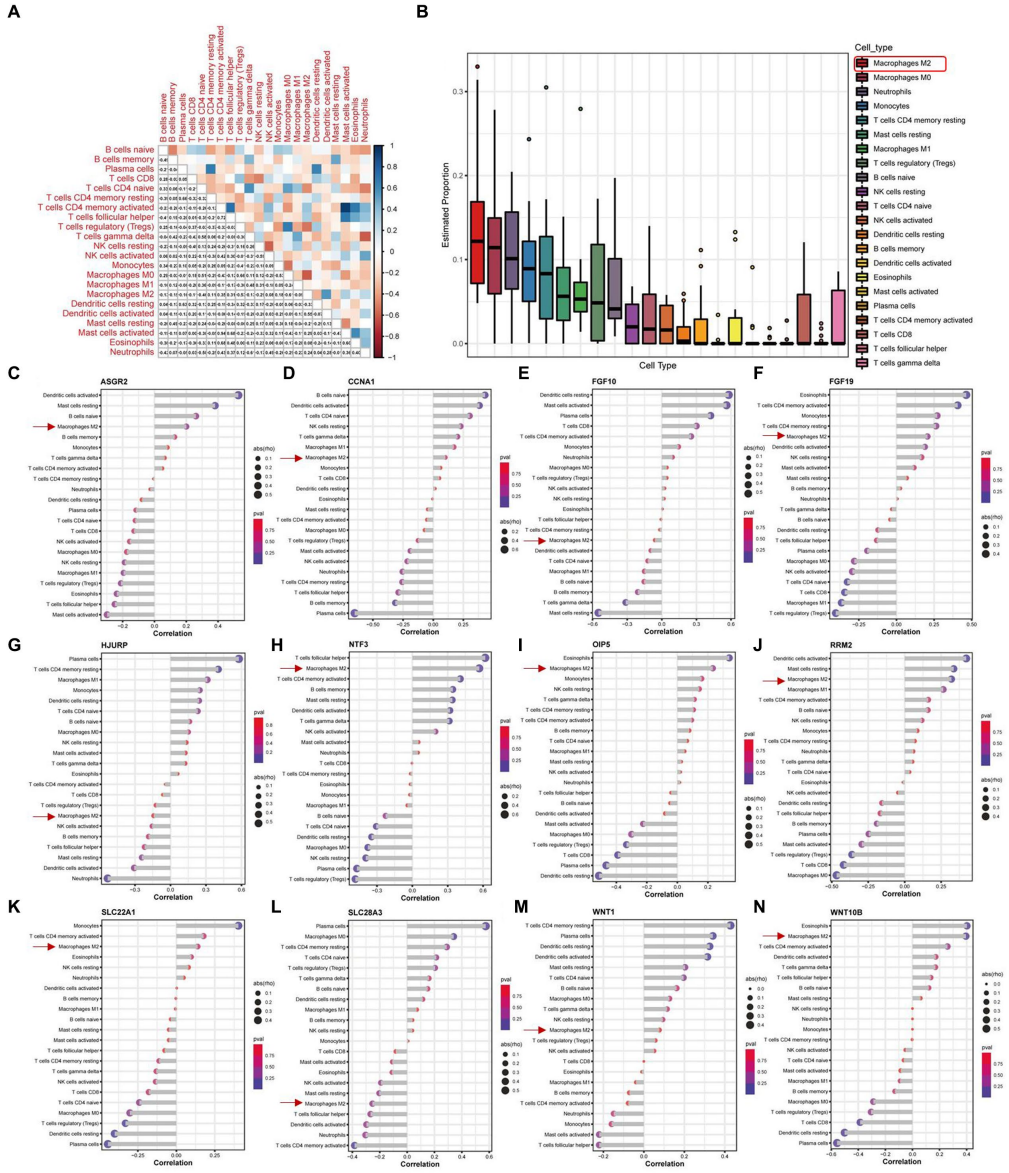
Immune infiltration analysis of PD samples and the expression of 12 key necroptosis related genes in various kinds of immune cells. **(A)** Correlation analysis between different species of immune cells. **(B)** The proportion of different immune cells in all samples. **(C–N)** The Correlation analysis between 12 key necroptosis related genes, including ASGR2, CCNA1, FGF10,FGF19, HJURP, NTF3, OIP5, RRM2, SLC22A1, SLC28A3, WNT1, and WNT10B, and various types of immune cells. Red square and arrows indicated the M2 macrophage.

We conducted an immuno-infiltration analysis using the expression matrix of PD samples in the GSE7621 dataset. The correlation between different species of immune cells and the proportion of different immune cells in all samples were presented in Figures 7A,B, respectively. These results suggested that the proportion of M2 macrophages was the highest. Besides, we further analyzed the correlations between the 12 above key necroptosis related genes in various types of immune cells, and the correlation coefficients and *p*-values were presented in the form of lollipop plots (Figures 7C–N). We could find that the expression of 9 genes (ASGR2, CCNA1, FGF19, NTF3, OIP5, RRM2, SLC22A1, WNT1, and WNT10B) were positively co-related to the function of M2 macrophage while 3 genes (FGF10, HJURP and SLC28A3) were negatively co-related to the function of M2 macrophage.

### 3.7. Validation the expression levels of 12 key necroptosis related genes by external dataset, toxin-induced injury of neuroblast model, and peripheral blood lymphocytes of PD patients

**Figure 8:**
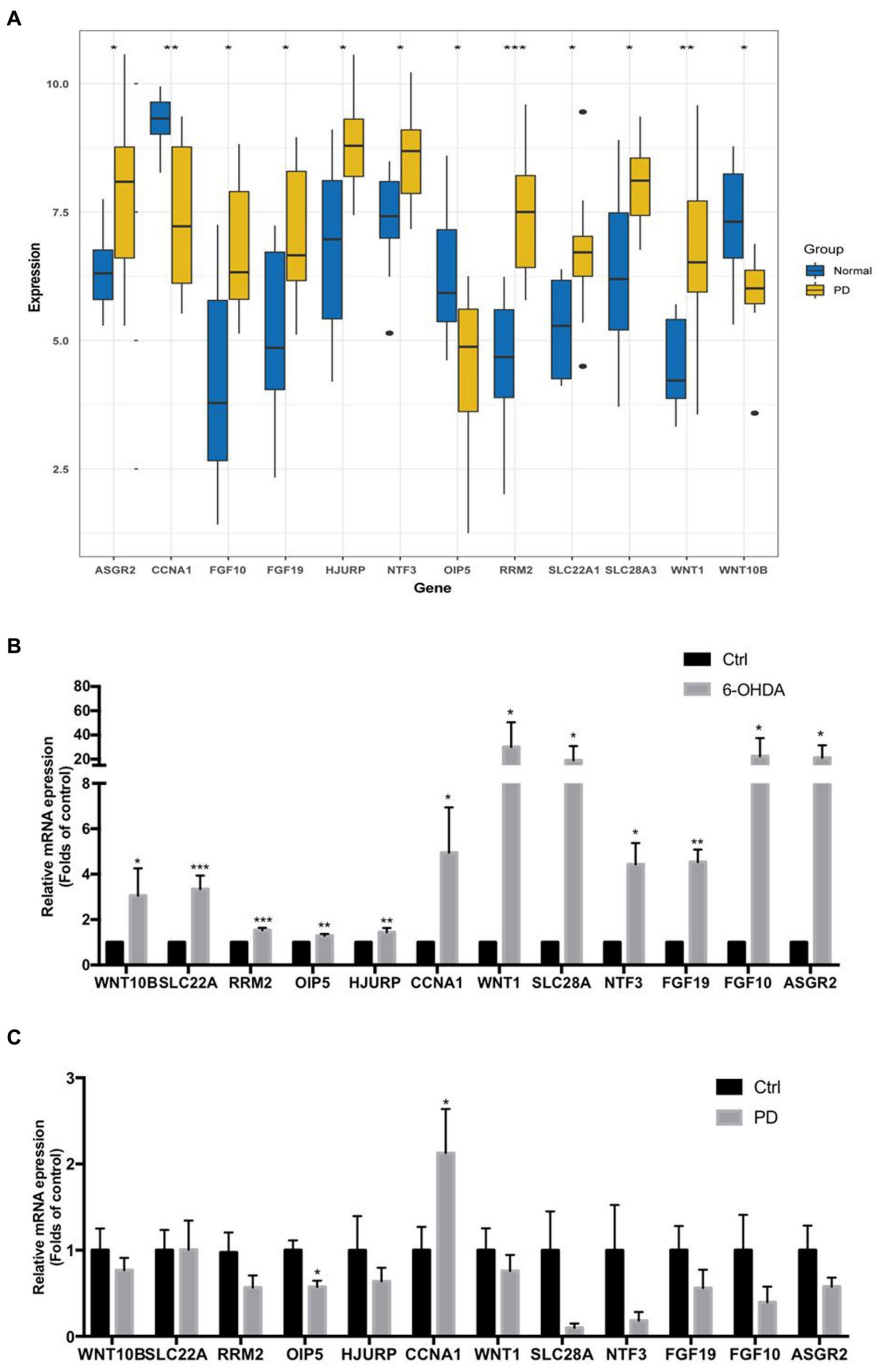
The mRNA expression levels of 12 key necroptosis related genes in external dataset, toxin-induced injury of neuroblast and peripheral blood lymphocytes of PD patients. **(A)** The expression matrix presented the expression of 12 key necroptosis related genes (ASGR2, CCNA1, FGF10, FGF19, HJURP, NTF3OIP5, RRM2, SLC22A1, SLC28A3, WNT1 and WNT10B) in both the control and PD groups from the dataset GSE20141. **(B)** The mRNA expression levels of above 12 key necroptosis related genes in toxin 6-OHDA-induced injury of neuroblast SH-SY5Y cell model. **(C)** The mRNA expression levels of above 12 key necroptosis related genes in peripheral blood lymphocytes of PD patients and health adults. *p < 0.05 vs. control group; **p < 0.01 vs. control group; ***p < 0.001 vs. control group.

As the gap analysis results from the external dataset GSE20141, the expression of ASGR2, FGF10, FGF19, HJURP, NTF3, RRM2, SLC22A1, SLC28A3 and WNT1 genes were significantly increased in PD samples, whereas the genes CCNA1, OIP5, and WNT10B were significantly decreased (Figure 8A). All these 12 genes were significantly upregulated in 6-OHDA treated SH-SY5Y cells (Figure 8B). However, CCNA1 was upregulated and OIP5 was downregulated in peripheral blood lymphocytes of PD patients (Figure 8C). All the above results were summarized in Table 2.

**Table 2:** Expression levels of 12 key necroptosis related genes in multiple models.

Genes	The full name of genes	GSE20141 dataset	Peripheral blood lymphocytes of PD patients	6-OHDA treated SH-SY5Y cell model
ASGR2	Asialoglycoprotein Receptor 2	+	n.s.	+
CCNA1	Cyclin A1	−	+	+
FGF10	Fibroblast Growth Factor 10	+	n.s.	+
FGF19	Fibroblast Growth Factor 19	+	n.s.	+
HJURP	Holliday Junction Recognition Protein	+	n.s.	+
NTF3	Neurotrophin 3	+	n.s.	+
OIP5	Opa Interacting Protein 5	−	−	+
RRM2	Ribonucleotide Reductase Regulatory Subunit M2	+	n.s.	+
SLC22A1	Solute Carrier Family 22 Member 1	+	n.s.	+
SLC28A3	Solute Carrier Family 28 Member 3	+	n.s.	+
WNT1	Wnt Family Member 1	+	n.s.	+
WNT10B	Wnt Family Member 10B	−	n.s.	+

Remark: +, significantly upregulation vs. control group; −, significantly downregulation vs. control group; n.s., no significant difference vs. control group.

## 4. Discussion

Parkinson’s disease (PD) has a widespread and significant negative impact on the motor function and life quality of patients. The current treatment methods significantly ameliorate its symptoms, but they cannot prevent its deterioration (Brocker et al., 2017). Although there are many studies focused on PD in recent decades, its pathogenesis is still not fully understood, which limited the development of its specific drugs. Necroptosis, which is a new form of regulated cell death and also exists in dopaminergic neurons, has been proved that it contributed to the pathological progression of PD (Liu et al., 2014). Conventionally, necrosis and apoptosis are two well-known forms of programmed death in injured neuronal cells. Recently, many researchers found that necroptosis is also a typical mode of neuronal cell death with the feathers of morphological changes in necrotic cells, activation of autophagy and energy-depleting (Imre, 2020). In addition, necroptosis is not the same as the traditional sense of necrosis (Carnevale et al., 2012). In the current study, we designed a comprehensive bioinformatic analysis and experimental validation strategy for identification of key necroptosis related genes in PD (Figure 1).

DEGs were extracted by gap analysis of dataset GSE7621, and followed by overlay of necroptosis genes (Figure 2). Consequently, 4 genes (UHRF1, GBE1, TRPC6 and TNIP3) were obtained and used for the guidance of cluster analysis (Figure 3) and enrichment analysis (Figure 4; Supplementary Tables S1–S4). In terms of molecular mechanisms, this study identified relevant molecular interactions through GO and KEGG enrichment analysis. GO enrichment analysis showed that positive regulation of cell proliferation, cell–cell signaling, lipid metabolic process, and wound healing are obvious in the biological participation processes (Figure 4A; Supplementary Table S1). KEGG enrichment analysis showed that neuroactive ligand-receptor interaction, calcium signaling pathway, and regulation of actin cytoskeleton are obvious (Figure 4B; Supplementary Table S2). Various related signaling pathways were also identified by both GSEA and GSAV enrichment analysis (Figures 4C-M; Supplementary Tables S3, S4). According to WGCNA analysis (Figure 5) and PPI network construction analysis (Figures 6A,B), 12 key necroptosis-related genes were identified, for example FGF10 and FGF19 which are the members of the FGFs family. FGF is a cell family signaling proteins closely related to neurodegenerative diseases. FGF and its receptor FGFR, which dramatically enhance the survival of dopaminergic neurons, play important roles in the development and maintenance of the heath nervous system as well as neuroinflammation (Chen et al., 2020). In cellular models of PD, FGF provides effective protection against the loss of dopaminergic neurons, promotes the development and survival of the nervous system, relieves neurological symptoms, and exerts neurotrophic activity in DA neurons (Liu et al., 2021). Despite to all these 12 key necroptosis related genes are positively related with the progression of PD, their internal relationship is not well known. We found that RRM2 and WNT1 were positively and negatively correlated with SLC22A1, while WNT10B presented positively correlation with both OIF5 and FGF19 (Figures 6C–G). These results indicated that these 12 necroptosis related genes are vital in the death of neurons and progression of PD, and the regulation network of these 12 key necroptosis genes are also complicate. Furthermore, the immune state in the brain of PD patient, such as neuroinflammation, also affects the neurodegeneration. Consistently, we also found the immune-inflammation changes in PD brain samples. As the results from immune infiltration analysis, many kinds of immune cells were active in PD samples in which M2 macrophage was the highest population of immune cell (Figures 7A,B). Moreover, the expression of 9 genes were positively correlated with differentiation and function of M2 macrophage while 3 genes were negative in these 12 key necroptosis genes (Figures 7C–N). M2 macrophage is a kind of anti-inflammatory phenotype in cardiovascular disease (Ma et al., 2020) while it promotes progression of cancer (Liu et al., 2019). Whether M2 macrophage enhanced the neurodegeneration in PD is not well elucidated. Thus, the immune state is really disrupted in the brain of PD patients and the immune cells, such as M2 macrophage, might also affect the expression of these 12 key necroptosis genes, resulting in the progression of neuron death.

In order to further verify the data, the mRNA expression levels of these 12 identified key necroptosis related genes were verified in both PD patients and 6-OHDA treated SH-SY5Y neuroblast model as well as another dataset GSE20141. Finally, we found that 3 genes were downregulated while 9 genes were upregulated in PD samples according to the DEGs analysis of dataset GSE20141 (Figure 8A). The mRNA expression of all these 12 genes were upregulated in 6-OHDA-treated SH-SY5Y cell model (Figure 8B), which was consistent with our current analysis. Although we conducted the immune infiltration analysis of PD brain samples which contributed to explore the immune state and expression of these 12 key necroptosis related genes in the brain of PD patients, the mRNA expression of these key necroptosis related genes in immune cells from peripheral blood were not known as well as their correlation. Thus, we collected the peripheral blood lymphocytes from both PD patients and control people, and detected the mRNA expression levels of these genes, which might be benefit for the clinical translation of our study. However, we only found that the mRNA expression level of CCNA1 was upregulated while OIP5 was downregulated in peripheral blood lymphocytes of PD patients (Figure 8C). As the summary of these genes expression listed in Table 2, the variability of gene expression results from peripheral blood lymphocytes of PD patients and the consistent of these genes expression in PD neuroblast cells indicated that neuron cell might mainly determine the genes expression levels of these 12 key necroptosis related genes in the brain. In addition, the total number of peripheral blood lymphocyte samples (6 PD + 6 control) might also limit the obtain of accurate result. Our study still needs further investigation in animal model, particularly the internal relationship of all these identified key necroptosis related genes.

Taken together, we could conclude that necroptosis and its associated inflammation play fundamental roles in the progression of PD and these identified 12 key genes might be served as new diagnostic markers and therapeutic targets for PD.

## Data availability statement

The raw data supporting the conclusions of this article will be made available by the authors, without undue reservation.

## Ethics statement

The studies involving human participants were reviewed and approved by Ethics Committee of Longhua Hospital, Shanghai University of Traditional Chinese Medicine. The patients/participants provided their written informed consent to participate in this study.

## Author contributions

CL and ZZ are jointly and comprehensively responsible for project design, experiment division, data analysis, manuscript preparation and submission. ZJ, SW, and QL complete experimental operation and data recording. YJ and HH gives technical guidance and consultation. YQ and ZZ participates in project design and provides funding support. All authors contributed to the article and approved the submitted version.

## Funding

This study was supported by National Natural Science Foundation of China (82074355 and 82174506) and Shanghai Municipal Health Commission (GWIV-28).

## Conflict of interest

The authors declare that the research was conducted in the absence of any commercial or financial relationships that could be construed as a potential conflict of interest.

## Publisher’s note

All claims expressed in this article are solely those of the authors and do not necessarily represent those of their affiliated organizations, or those of the publisher, the editors and the reviewers. Any product that may be evaluated in this article, or claim that may be made by its manufacturer, is not guaranteed or endorsed by the publisher.

## Footnotes

^1^ https://www.genecards.org/

^2^ https://david.ncifcrf.gov/

^3^ https://string-db.org/

^4^ https://www.r-project.org/
