# Supplementary material for: Identification of necroptosis-related genes in Parkinson’s disease by integrated bioinformatics analysis and experimental validation
Source: Front Neurosci. 2023 May 22;17:1097293. doi: 10.3389/fnins.2023.1097293 (PMC10239842; doi:10.3389/fnins.2023.1097293)
Supplement: Supplementary file 1 [file Data_Sheet_1.docx]

**Table S1 The top ten IDs of BP according to the results of GO enrichment analysis of necroptosis-related genes.**

| ONTOLOGY | ID | Description | PValue | Count |
| --- | --- | --- | --- | --- |
| BP | GO:0007267 | cell-cell signaling | 1.06E-04 | 10 |
| BP | GO:0006629 | lipid metabolic process | 2.88E-04 | 9 |
| BP | GO:0008543 | fibroblast growth factor receptor signaling pathway | 0.00729 | 4 |
| BP | GO:0042060 | wound healing | 0.008867 | 5 |
| BP | GO:0007187 | G-protein coupled receptor signaling pathway | 0.01574 | 4 |
| BP | GO:0008284 | positive regulation of cell proliferation | 0.029882 | 10 |
| BP | GO:0051482 | positive regulation of cytosolic calcium ion concentration | 0.030812 | 3 |
| BP | GO:0035025 | positive regulation of Rho protein signal transduction | 0.030812 | 3 |
| BP | GO:0010951 | negative regulation of endopeptidase activity | 0.03435 | 5 |
| BP | GO:0003406 | retinal pigment epithelium development | 0.03981 | 2 |

**Table S2 The top five IDs of pathway according to the results of KEGG enrichment analysis of necroptosis-related genes.**

| ONTOLOGY | ID | Description | PValue | Count |
| --- | --- | --- | --- | --- |
| KEGG | hsa04080 | Neuroactive ligand-receptor interaction | 0.005765 | 10 |
| KEGG | hsa04020 | Calcium signaling pathway | 0.006399 | 8 |
| KEGG | hsa04810 | Regulation of actin cytoskeleton | 0.014927 | 7 |
| KEGG | hsa00561 | Glycerolipid metabolism | 0.018443 | 4 |
| KEGG | hsa04918 | Thyroid hormone synthesis | 0.031595 | 4 |

**Table S3 The top five IDs the up- and down-regulated pathways according to** **the results of GSEA enrichment analysis of necroptosis-related genes.**

| Description | setSize | enrichmentScore | NES | PValue |
| --- | --- | --- | --- | --- |
| KEGG_ALPHA_LINOLENIC_ACID_METABOLISM | 15 | 0.690131 | 1.810236 | 0.003883 |
| KEGG_ETHER_LIPID_METABOLISM | 29 | 0.584976 | 1.798945 | 0.001996 |
| WP_TRIACYLGLYCERIDE_SYNTHESIS | 23 | 0.605011 | 1.747378 | 0.00396 |
| REACTOME_RECOGNITION_AND_ASSOCIATION_OF_DNA_GLYCOSYLASE_WITH_SITE_CONTAINING_AN_AFFECTED_PURINE | 44 | 0.503895 | 1.705788 | 0.006 |
| REACTOME_PLASMA_LIPOPROTEIN_ASSEMBLY_REMODELING_AND_CLEARANCE | 70 | 0.441818 | 1.641828 | 0.005941 |
| WP_SYNAPTIC_VESICLE_PATHWAY | 51 | -0.63569 | -2.22711 | 0.001976 |
| REACTOME_NEUROTRANSMITTER_RELEASE_CYCLE | 51 | -0.61078 | -2.13983 | 0.001976 |
| REACTOME_PRESYNAPTIC_DEPOLARIZATION_AND_CALCIUM_CHANNEL_OPENING | 11 | -0.83765 | -2.05204 | 0.002075 |
| REACTOME_GABA_SYNTHESIS_RELEASE_REUPTAKE_AND_DEGRADATION | 19 | -0.69285 | -1.97965 | 0.00409 |
| REACTOME_NEUROTOXICITY_OF_CLOSTRIDIUM_TOXINS | 10 | -0.81233 | -1.957 | 0.002012 |

**Table S4** **Results of GSVA enrichment analysis of necroptosis-related genes.**

| ID | logFC | PValue |
| --- | --- | --- |
| REACTOME_THYROXINE_BIOSYNTHESIS | 0.645118 | 1.17E-05 |
| GO_COBALAMIN_TRANSPORT | 0.698664 | 1.75E-05 |
| GO_REGULATION_OF_METANEPHRIC_GLOMERULUS_DEVELOPMENT | -0.80306 | 2.70E-05 |
| GO_RESPONSE_TO_INACTIVITY | 0.554813 | 3.28E-05 |
| GO_POSITIVE_REGULATION_OF_METANEPHRIC_GLOMERULUS_DEVELOPMENT | -0.80687 | 7.56E-05 |
| GO_CYTOCHROME_B5_REDUCTASE_ACTIVITY_ACTING_ON_NAD_P_H | 0.673376 | 0.000102 |
| GO_AGGRESSIVE_BEHAVIOR | 0.677764 | 0.000177 |
| GO_BRUSH_BORDER_ASSEMBLY | 0.647769 | 0.000191 |
| DELASERNA_TARGETS_OF_MYOD_AND_SMARCA4 | 0.585091 | 0.000261 |
| GO_REGULATION_OF_SYSTEMIC_ARTERIAL_BLOOD_PRESSURE_BY_VASOPRESSIN | 0.739123 | 0.00033 |
